# One swallow does not a summer make: Twenty years of challenges and achievements of family medicine in Mozambique

**DOI:** 10.4102/phcfm.v14i1.3335

**Published:** 2022-02-03

**Authors:** Yolanda Sabino, Palmira A. Francisco, Ofelia da Conceição Rambique, Leyani A. Chavez Noya, Armando J. Bucuane, Adelson G. Jantsch

**Affiliations:** 1 Departament of Family and Community Medicine, Eduardo Mondlane University’s Health Center, Polana Caniço General Hospital, Maputo, Mozambique; 2 General Secretary at the College of Family and Community Medicine of Mozambique, Maputo, Mozambique; 3 Health Sciences Department, Faculty of Medicine, Universidade Unilurio, Nampula, Mozambique; 4 Presidency at the College of Family and Community Medicine of Mozambique, Maputo, Mozambique; 5 Foundation for Scientific and Technological Development in Health (Fiotec), Rio de Janeiro, Brazil

**Keywords:** family medicine, primary health care, developing countries, Mozambique, healthcare workforce

## Abstract

After 20 years of hard work, family medicine (FM) is flourishing in Mozambique, but the challenges are immense in a context of multiple health needs. This study aimed to describe strengths, opportunities, weaknesses and threats of the current scenario that can influence the development of FM and primary health care (PHC) in Mozambique. Case study of a series of virtual world-café meetings using the World Health Organization’s Operational Framework for primary health care as a theoretical model. There is a young generation of Family Physicians (FPs) eager to improve PHC in Mozambique – a result of the reactivation of the Maputo Residency Programme and the creation of the Mozambican College of FP in 2010. The current Ministry of Health has taken this agenda forward, inviting medical societies (including FM) to jointly design plans to expand training of human resources for healthcare. This plan aims to create new training sites in five different provinces hoping that it will increase the number of FP in remote areas, fixing the unequal distribution of specialists in the country. The small number of FP practicing today and the limited financial resources of the National Government are important threats to this plan. We have many strengths already conquered and the current situation opens an opportunity for the expansion of FM in Mozambique. Hopefully, it will help PHC in our country move from verticalised and selective health programmes towards a more comprehensive, efficient and person-centred care.

## Introduction

Only eight family physicians (FPs) have been trained in Mozambique. Ten, if we count two Mozambicans who graduated outside the country. Given the size of our population (31.3 million in 2020), this number is extremely small and we certainly still have a long way to go before we can say that our specialty impacts the health status of our population.^1^

This number could have been higher if it was not for so many difficulties experienced in the short life of family medicine (FM) in Mozambique. The first training programme in FM began in 2000 at the *Hospital Geral Polana Caniço* in Maputo, with the Spanish Cooperation taking care of the operations and academic management. In 2009, the programme was closed, having trained only four FPs. Only in 2015 did the programme resume its activities, with one resident starting his training that year and two more in 2016. There are currently two programmes in the country: one in Maputo and another in Nampula, which has not received any residents yet, despite having the accreditations and capacity to train new FPs.

Despite this uncertain and timid beginning, the current national scenario is very positive for the growth of FM and primary healthcare (PHC). To keep taking the right steps, we need to analyse the current political scenario and the health context in which we live. This case study is the result of a series of virtual world-café meetings amongst the authors using the World Health Organization’s Operational Framework for Primary Health Care as a theoretical model.^2^ It aims to describe the strengths, opportunities, weaknesses and threats of the current scenario that can influence the development of FM and PHC in Mozambique.

## Current context

Mozambique is a country with many social, economic and health needs. The country ranks 22nd on the Fragile States Index list^3^ and is experiencing an incomplete epidemiological transition scenario,^4^ where the burden of morbidity resulting from infectious diseases competes with an increasing prevalence of non-communicable chronic conditions. With just over 30 million inhabitants and a GDP per capita of US$467, we still have high maternal (289 per 100 000 live births) and infant mortality rates (74.2 per 1000 live births) and a prevalence of HIV of 11.5%, greater than most chronic non-communicable diseases. To deal with this high burden of morbidities, we have an extremely small ratio of doctors per population – just one doctor for every 10 000 inhabitants.^1^

As a result of the comprehensive training, the work of the family doctor, when integrated with other professionals in the PHC team can be decisive in meeting the health needs of the population in Mozambique.^5^ Certainly, the skills developed over the four years of training makes them more capable of meeting the demands of the population. On the other hand, this same training makes local authorities see these FPs as skilled professionals who can occupy positions in the Ministry of Health (MoH) or provide managerial support in public services. Even when such duties are intermittent, the hours available for teaching and taking care of patients is reduced. In addition, professional recognition is not accompanied by financial compensation for any medical specialty in Mozambique.

Despite the quality training that we seek to offer to our residents, much of what is learned cannot be applied in clinical practice. Community health clinics lack the most basic resources and supplies for comprehensive patient care. This hinders the population’s confidence in primary care clinics, making patients and families seek care in more resourced hospital services.

### Considerations for the near future

There is a growing movement of young FPs in Mozambique, as a result of the reactivation of the Maputo Residency Programme and the creation of the Mozambican College of FP in 2010. Despite the optimism and engagement, this group is still too small to generate political changes and modify practices in universities, health services and public administration. Fortunately, the current MoH’s agenda has been trying to fix the lack of trained human resources in our country, inviting medical societies to jointly design a plan for expanding residency training for all medical specialties with FM occupying a prominent seat at this table of negotiations.

This initiative will create new training sites in remote areas far from the capital, following successful experiences from other countries. In this way, instead of attracting young doctors from small cities to be trained in Maputo, the necessary resources will be deployed to 10 healthcare facilities in five provinces of the country, improving the quality of these services. The aim is to make future generations of FPs settle in the places where they were originally trained, increasing the number of specialists in areas that have no FP today and improving their distribution throughout the country.

This MoH initiative represents the beginning of a paradigm shift in medical education in our country, which still values tertiary hospitals and specific medical specialties as more important than the work of generalists in the community. Despite this favourable political scenario, there are major challenges to achieve this goal. Initially, the aim was to train 750 new FPs by 2025, but this number proved to be impractical because of structural and financial shortcomings. Currently, 10 health facilities in five provinces are listed as potential sites to become new residency programmes in FM. If that happens, in 2022 we will have 60 vacancies for residents spread throughout our country: 9 in Maputo province, 12 in Sofala, 12 in Zambézia, 12 in Tete and 18 in Nampula, in addition to the five vacancies in Polana Caniço.

New preceptors must be hired to teach and practice in these centers. The number of FPs about to graduate will not be enough to meet this demand. In 2022, five new FPs will graduate, but there will be no one graduating in 2023. Only in 2024 and 2025 we will have eight and seven new specialists graduating, respectively. In the short term, it is almost certain that Mozambique will need to rely on the Cuban International Cooperation to remediate this shortage of trained preceptors.^6^

In order to strengthen this initiative, it will be crucial to face another major challenge in the short term: building and validating a competency-based curriculum for FM in Mozambique. This will help us to create objective parameters to guide both the training and practice of FP and make the expansion of residency training in FM uniform amongst all six training sites.^7^

We also need to make our specialty more present and active in medical schools so that this transformation is sustainable in the long-term. Students have very little contact with FM whilst studying at medical school. Their contact with patients in the community happens under the supervision of nurses and technicians (technical professionals responsible for most of the primary care delivered in Mozambique) and takes place in health facilities where they get contact with health programmes focused on maternal and child care, HIV and AIDS, tuberculosis and vaccination. Students rarely have the opportunity to follow medical appointments during these visits and when this happens, it is not a FP running the consultation.

The Eduardo Mondlane University Health Center is the training site for residents in FM in Maputo. It serves students, university employees, employees’ families and residents of the neighborhood. Unfortunately, medical students only have contact with what we call community health. This discipline presents a simplistic view of what PHC and FM really are. Medical students and other fellow professors at the university end up discrediting the specialty, seeing it as lacking glamour and excitement.^8^

This misconception about the work of FPs needs to be ^changed^ and it relies on the efforts we have been making, seeking greater integration with other medical specialties and other healthcare professionals.^9^ In addition, we need to improve teaching and learning experiences in FM at the residency programme and medical school. If we succeed in this endeavour the next generations will fully experience FM and will start to see primary care as a desirable place where medicine can be taught and learnt by any healthcare student.

This new generation of FPs will have a lot of work ahead, advocating for the specialty with politicians and decision-makers. The momentum created by the resurgence of the residency programmes in 2015, the promotion of this new generation of FP and the current political scenario creates a favourable environment for the sustainable expansion of FM.^8^ Collaboration with colleagues from abroad, international internships, continuing medical education, faculty development activities and capacity building for research in PHC are key elements for FM to flourish in Mozambique.^10^ Finally, optimisation studies would be helpful to better plan the investment of the scarce financial resources we have.

The information described here is summarised in Table 1 presenting the strengths, opportunities, weaknesses and threats of the current scenario of FM and PHC in Mozambique, categorised according to the World Health Organization’s 14 levers for the development of PHC.

**TABLE 1:** Strengths, opportunities, weaknesses and threats of the current scenario of family medicine and primary health care in Mozambique categorised according to the World Health Organization’s 14 levers for the development of primary healthcare.

WHO	Strengths and opportunities	Weaknesses and threats
Political commitment and leadership	Current MoH promoting medical training in PHC and FM. Engaging with local leaders in FM.	Poor general understanding by other medical disciplines and general population about family medicine and primary care.
	Family medicine and PHC are a priority in the MoH’s agenda.	
Governance and policy frameworks	Capacity building of community health workers to revitalise the local community health subsystem.	Public health managers still attached to a health service paradigm focused on hospitals and specialists.
	Expansion of specialised training in all medical areas as a priority on the ministerial agenda.	Family physicians are not taking part in the community health subsystem reform, only public health experts.
Funding and allocation of resources	There are many non-government organisations financing and providing primary care in the country.	Lack of financial incentives to attend the residency programmes and to subsidise tutors.
		Most of the funds allocated to hospital expenditure.
Engagement of communities and other stakeholders	Long-standing ties with Cuban Cooperation, strengthening the specialty.	Traditional healers, midwifes, FP and other stakeholders in the community working with no integration. Still missing an integrated leadership.
	Support from Cuban Cooperation in the provision of teachers of FM, strengthening the team of faculties.	
Models of care	Competency-based curriculum for MFC almost completed.	Most of the primary care is provided by community health workers or technicians.
		Family physicians still not fully integrated in the PHC system because of shortage of specialists.
Primary health care workforce	Community-based primary care performed by healthcare team, with technicians, nurses, dentists and generalists.	Shortage of physicians, nurses, dentists and all medical specialties in the country.
	Traditionally, physicians are primary care providers in the community, not only in hospital setting.	Small contingent of FP and only one residency programme receiving students.
	We have a consistent growth in the demand for residence vacancies in MF.	Few vacancies for the current demand size.
	Young residents are very motivated.	Lack of tutors to train the next generations.
Physical infrastructure	There are many community health centers across the country, and the MoH is constantly building new ones.	Poor physical structure. Lack of adequate health supplies and tools to respond to population demand.
Medicines and other health products to improve health	When available, medicines and procedures are free of charge for all patients.	Ministry of health faces challenges to provide essential medicines most of the time in all health units and in all levels of healthcare.
Engagement with private-sector	Many private health services all over the country.	The private system is totally isolated from the public system, there is neither coordination nor integration between the two systems. Although they are the same health professionals who provide both private and public services, especially specialised professionals.
Purchasing and payment systems	There is a different salary between general practitioners and specialised doctors.	Little financial return for those who specialise.
Digital technologies for health	Online courses for medical students and residents during the COVID-19 pandemic.	There is no use of health technology in the healthcare system.
Systems for improving the quality of care	There is a great effort by the MoH to train more health professionals from all areas and at all levels of healthcare to improve the quality of care provided in Mozambique.	There is no financial support to improve the quality of service in the health centres.
	Some NGO’s promote specific quality improvement interventions focused in specific healthcare issues – that is, tuberculosis and HIV.	Lack of training in quality improvement skills and tools.
		Mainly short-term quality improvement initiatives.
Primary health care-oriented research	National Institute of Health has a professional career for those who want to become medical researchers.	Lack of basic research skills.
	New generation of FPs interested in doing research in FM and PHC.	Lack of support for research projects in the community.
	Residents of FM rotate at the Polana Caniço Hospital’s research unit.	Lack of financial support for publications.
Monitoring and evaluation	Healthcare data generated everyday by healthcare providers.	Real-world data generated at the community healthcare center is poorly analysed and usually does not influence changes in service delivery.
	Local managers promote discussions with healthcare providers using real-world generated at the community health care centre.	Lack of data driven decision making.
	Data inputted in paper sheets are transferred to a computer system linked directly to the MoH (Sistema SISMA).	

*Source*: Adapted by the authors from the World Health Organization and the United Nations Children’s Fund (UNICEF). Operational framework for primary health care: Transforming vision into action [homepage on the Internet]. Geneva, 2020 [cited 2021 Aug 29]; 132 p. Available from: https://www.who.int/rehabilitation/CallForAction2.pdf?ua=1

FM, family medicine; PHC, primary healthcare; WHO, World Health Organization; MoH, Ministry of Health; FP, family physician; NGO, non-governmental organisation; HIV, human immunodeficiency virus.

## Conclusion

As the saying goes: ‘One swallow does not a summer make’. Perhaps eight swallows can, with small victories each day, make PHC in Mozambique less based on verticalised healthcare programmes and more based on comprehensive, efficient and person-centred care.
